# Poisonous Milk

**Published:** 1858-05

**Authors:** 


### POISONOUS MILK.

Action is the universal law of animal life. There is not a living thing, whether insect, or bird, or beast, that will not pine and fall away, and perish, under bodily restraint. Man himself is no exception to the world-wide ordinance. The flesh of no pent-up fowl or brute ought to be eaten, because it is diseased flesh. No wonder that gourmands luxuriate on "wild game;" the meat is not only more tender, and sweet, and juicy, but it is healthy flesh.

Many families are considered fortunate, who can afford to keep a cow in cities and large towns. But however various may be their food, and abundant, and however clean their stalls, they inevitably become diseased, and within a very short time too, unless they can walk about in the open air, and crop something from the surface of the earth. Hence the milk of stabled cows becomes putrid much sooner than that of a pastured animal; for the microscope discovers minute globules of corrupted matter, floating through this stabled milk. Just imagine, reader, a few drops of the yellow matter of a sore, stirred in among the milk, which, from silver pitchers, you pour into your morning coffee!

The reason of this is, a confined cow gets little or no lime from the food which she eats. This lime she gets from the green grass itself, and an additional quantity in the shape of dust, which settles on the grass, or sticks to some of the roots of the grass, which sometimes are pulled up in her browsings. If, therefore, a cow must be stabled, a handful of bone-dust should be mixed with her food every day. A cow will very soon become consumptive if closely confined; and infants might as well use the milk of diseased mothers, as that of diseased cows.

These are facts about which we presume there can be no dispute, and we consider ourself as the author of a benefaction to any family whom we can induce to use the "*Condensed Milk*" which is begun to be introduced into New York, which is nothing more than milk deprived of a great part of its water, and left thicker than the thickest cream.

As it leaves no sediment, it is proof so far that it is pure. Two teaspoonfuls of it whiten a cup of tea or coffee as much as half a cupful of boiled milk, thus saving a family that troublesome process.

Another advantage is, it will remain perfectly good for weeks, in summer time, if kept in an ice-chest; and as long in winter, if kept in a dry airy place, not cold enough to freeze. Thus, by taking it once a week, the daily noise of milkmen and the trouble of attending to them, is wholly got rid of; and being brought from the interior of New England, where there are no distilleries to slop-feed the cows, but where it is known to be a farming neighborhood, there is every guarantee that we are using the milk of farm-house cows. It is afforded now, at rather a less cost than common city milk. The only imposition to which we are liable is in the thickness of this milk; and we hope the proprietors will, in time, sell it by weight. *Now*, an honest pint weighs just twenty-one ounces. Twenty years hence, that weight will be a fable, and will have dwindled down to about one-half. But, during MR. BORDEN'S lifetime, we had better all hurry up, and enjoy it in its true *gravity*.

This testimony to the value and the virtues of "Condensed Milk" is borne without the "knowledge or consent" of the proprietors; but from the conviction, from long use of it, that it is one of the most beneficent domestic luxuries which we have ever had occasion to notice in this Journal.

As a nutriment in consumptive diseases, it is, in our estimation, more efficient than cod-liver oil, for we know that consumptives need nutrition above all other things—the want of it is *the* thing which prevents recovery. And two other things we know: Pure milk is the only article in nature which has in it all the elements of nutrition; it has the element of heat, of repair, and of growth, while cod-liver oil has only the calorific element, and keeps us warm, nothing more; it gives no enduring strength; the patient gets heavier, but he gets no stronger, no more long-winded. It is wonderful that medical men have not had their attention directed to this most important distinction. As long as a consumptive person is getting no stronger, he is getting no better, however more favorable other symptoms may be growing. Thus it is that cod-liver oil, affording only the elements of heat, and milk giving both heat and repair, can never be a substitute for it.

But are consumptive people to suppose that, by drinking pure milk or cream abundantly, they are going to get well without consulting a doctor? The person who attempts it will die very much sooner, because any one living largely on milk will soon become costive, or derange his digestion, and then strength declines. But if an experienced physician can superintend the case, to keep the liver and bowels in proper condition, and will judiciously arrange the exercises of the patient, in a manner best calculated to digest a previous meal, and create a vigorous appetite for another, and do this for three meals a day, then the chances for protracting life in considerable comfort, or eradicating the disease, are manifold greater than by any other method hitherto devised.

